# Editorial and Miscellaneous

**Published:** 1864-05

**Authors:** 


### EDITORIAL AND MISCELLANEOUS.

*Etherization Followed by Death.*—At the meeting of the Imperial Society of Medicine in Lyons, on July 20, M. Chassagny communicated the case of a lady, aged 40, to whom æther was administered previously to the removal of an urethral polypus and two sebaceous cystic tumors on the head. Thirty *grammes* of æther (rather less than an apothecary's ounce) were used; but the anæsthesia produced was incomplete, and the patient was aware that the operations were being performed. The administration of the anæsthetic was not pushed further, because the stage of excitement did not manifest itself, and because, on the contrary, general coldness and slowness of the pulse were present. On the completion of the operation, which occupied a quarter of an hour, vomiting set in; the coldness increased, and was accompanied by clammy sweats; and the patient had convulsions, attended with foaming at the mouth. The attack passed away in a few moments, but soon returned with equal intensity. After the fourth attack, the patient died. M. Chassagny considered that the patient had died of eclampsia induced by etherization, which was thus the indirect cause of death.—*Brit. Med. Jour.* from *Gaz. Med. de Lyon*, 16 October, 1863.

This accident occurred but a few days before our visit to the city of Lyons, and was then the topic of conversation in all circles. When it is remembered that the French seldom administer anæsthetics to produce complete loss of consciousness, that the agent is as carefully prepared there as in any part of the world, that patients to whom it is administered are usually selected with care,—and still that fatal results follow its use there as elsewhere, the profession may well pause, as they do at present, to enquire, whether any means may be adopted to obviate, if not absolutely, at least the frequent occurrence of these tragic results. Doubtless the danger depends in part upon the impurity of the specimen used, but quite as much depends upon the incautious and too rapid administration.

In our use of anæsthesia, which has been somewhat extensive and frequent, we have yet to meet the first alarming occurrence. We have believed that *rapid* introduction of the agent into the system would account for the dangerous symptoms that sometimes so suddenly and unexpectedly arise.

This case demonstrates also that the use of æther, like chloroform; is not unattended with danger, when care is not exercised in its administration.

---

*The American Journal of Ophthalmology*.—This Journal commences its second volume as a quarterly. It is edited by Julius Homberger, M. D., New York. It contains much that is of great interest to the general practitioner, while to the oculist, a publication like this, must be indispensable. We reproduce in our present issue the article on the application of Atropine, by the editor. We commend the Journal to the profession, especially to those who devote themselves to the treatment of Ophthalmic diseases.

---

McMunn's Elixir of Opium.—*Does it possess any superiorty over other preparations of Opium?*—A correspondent in the *Philadelphia Med. and Surg. Reporter*, for February, gives the original formulary for making this preparation, so much vaunted as containing all the good qualities of the drug without any of its deleterious properties. Based on the testimonial of J. R. Chilton, M. D., the advertisements in the leading Medical journals of the country, the prescriptions of eminent physicians, it has become exceedingly popular, yielding immense profits to the manufacturer.

In the *N. Y. Medical Independent*, Prof. Percy has demonstrated that the "Elixir is nothing more than a solution of impure morphia," and from careful observations, that it possesses no property giving it any superiority over other preparations of opium.

We feel obliged for these communications, as we have no doubt the profession will, for they prove what we have long believed to be the fact in regard to this preparation; in experimenting with it, where we expected less cerebral disturbance —or less nausea, or less constipation,—from a given anodyne effect, than would follow the use of the ordinary preparations of opium, all of which are claimed for it, we have uniformly been disappointed.

It might be an interesting inquiry, how members of the regular profession, in good standing, can justify themselves in recommending and prescribing this—a secret remedy, as many have done, which was foisted into notice in as objectionable a manner as many of the quack nostrums which load the shelves of the druggists throughout the country—while theoretically, they are so exceedingly sensitive about the least violation of the "Code." Medical men should desert their standard, "*Nec prece nec pretio*."

